# Supplementary material for: Angiopoietin-2 in white adipose tissue improves metabolic homeostasis through enhanced angiogenesis
Source: eLife. 2017 Mar 29;6:e24071. doi: 10.7554/eLife.24071 (PMC5391203; doi:10.7554/eLife.24071)
Supplement: Supplementary file 1. — DOI: http://dx.doi.org/10.7554/eLife.24071.021 [file elife-24071-supp1.docx]

**Supplementary Files**

**Supplementary File 1. Primer sequences for qPCR**

| **Protein** | **Gene** | **Forward (5’ -3’)** | **Reverse (5’ -3’)** |
| --- | --- | --- | --- |
| GAPDH | *Gapdh* | TGTGAACGGATTTGGCCGTA | ACTGTGCCGTTGAATTTGCC |
| ANG-2 | *Angpt2* | CCAACTCCAAGAGCTCGGTT | CGGTGTTGGATGACTGTCCA |
| VEGF-A | *Vegfa* | GGAGATCCTTCGAGGAGCACTT | GGCGATTTAGCAGCAGATATAAGAA |
| TIE2 | *Tek* | AAGCAACCCAGCCTTTTCTC | TGAGCATTCTCCTTTGGAC |
| CD31 | *Pecam1* | AAGCAACCCAGCCTTTTCTC | CGACAGGATGGAAATCAACAA |
| TEM8 | *Antxr1* | TGTTCAGGGGATACTTGGCT | GAGTGTCTGTGATGAGGCCA |
| ANGPTL3 | *Angptl3* | CAGACCGTGGAAGACCAATA | GAAATTTCTGTGGGTTCTTGAAT |
| ANGPTL4 | *Angptl4* | GCACCTAGACCATGAGGTGG | GGAACAGCTCCTGGCAATC |
| ANGPTL8 | *Angptl8* | CCCTCAATGGCGTGTACAGA | CCACCTGAATCTCCGACAGG |
| F4/80 | *Adgre1* | TGACTCACCTTGTGGTCCTAA | CTTCCCAGAATCCAGTCTTTCC |
| IL-6 | *Il6* | CCAGAGATACAAAGAAATGATGG | ACTCCAGAAGACCAGAGGAAAT |
| CD115 | *Csf1r* | TGTCATCGAGCCTAGTGGC | CGGGAGATTCAGGGTCCAAG |
| CD11b | *Itgam* | GGCTCCGGTAGCATCAACAA | ATCTTGGGCTAGGGTTTCTCT |
| LY6C1 | *Ly6c1* | ATTGAGACTTCCTGCCCAGC | GATCCCTGATTGGCACACCA |
| *CD206* | *Mrc1* | TGTGGTGAGCTGAAAGGTGA | CAGGTGTGGGCGCAGGTAGT |
| IL-10 | *Il10* | GCTCTTACTGACTGGCATGAG | CGCAGCTCTAGGAGCATGTG |
| CD301 | *Clec10a* | TGAGAAAGGCTTTAAGAACTGGG | GACCACCTGTAGTGATGTGGG |
| IFNγ | *Ifng* | TCAAGTGGCATAGATGTGGAAGAA | TGGCTCTGCAGGATTTTCATG |
| TNFα | *Tnfa* | GAGAAAGTCAACCTCCTCTCTG | GAAGACTCCTCCCAGGTATATG |
| NOS2 | *Nos2* | CAGAGGACCCAGAGACAAGC | TGCTGAAACATTTCCTGTGC |
| UCP-1 | *Ucp1* | TCTCAGCCGGCTTAATGACTG | GGCTTGCATTCTGACCTTCAC |
| COL1A1 | *Col1a1* | GTGCTCCTGGTATTGCTGGT | AAGGACCATCCCACTGTCTG |
| COL3A1 | *Col3a1* | GGGTTTCCCTGGTCCTAAAG | CCTGGTTTCCCATTTTCTCC |
| COL6A1 | *Col6a1* | GATGAGGGTGAAGTGGGAGA | CAGCACGAAGAGGATGTCAA |
| LOX | *Lox* | CCACAGCATGGACGAATTCA | AGCTTGCTTTGTGGCCTTCA |
| HIF-1α | *Hif1a* | CAAGATCTCGGCGAAGCAA | GGTGAGCCTCATAACAGAAGCTTT |
| GLUT1 | *Slc2a1* | CCTGTCTCTTCCTACCCAACC | GCAGGAGTGTCCGTGTCTTC |
