# Supplementary material for: Angiopoietin-2 in white adipose tissue improves metabolic homeostasis through enhanced angiogenesis
Source: eLife. 2017 Mar 29;6:e24071. doi: 10.7554/eLife.24071 (PMC5391203; doi:10.7554/eLife.24071)
Supplement: Supplementary file 2. — DOI: http://dx.doi.org/10.7554/eLife.24071.022 [file elife-24071-supp2.docx]

**Supplementary Files**

**Supplementary File 2. Statistical Information**

| **Figure** | **N(sample size)** | **Statistical test method** | ***p*-value** |
| --- | --- | --- | --- |
| Figure 1B | Adipo-rtTA: 7  Adipo-ANG2: 7 | Unpaired *Student’s t* test | sWAT: *p*=0.0007  eWAT: *p*=0.0002  BAT: *p*<0.0001 |
| Figure 1C | Adipo-rtTA: 5  Adipo-ANG2: 5 | Unpaired *Student’s t* test | *p*=0.0301 |
| Figure 2A | Adipo-rtTA: 6  Adipo-ANG2: 6 | Unpaired *Student’s t* test | *Vegfa:* *p=*0.0005  *Tek: p<*0.0001  *Pecam1: p=*0.0103  *Antxr1: p<*0.0001 |
| Figure 2C | Adipo-rtTA: 6  Adipo-ANG2: 6 | Unpaired *Student’s t* test | *p*<0.0001 |
| Figure 3A | Adipo-rtTA: 10  Adipo-ANG2: 10 | Two-way analysis of variance (ANOVA) followed by a Bonferroni post-test to compare replicate means in each time point | Week 4: *p=*0.0375  Week 5: *p=*0.0036 |
| Figure 3B | Adipo-rtTA: 10  Adipo-ANG2: 10 | Unpaired *Student’s t* test | Fat: *p=*0.0013  Lean: *p=*0.0010 |
| Figure 3C | Adipo-rtTA: 10  Adipo-ANG2: 10 | Two-way analysis of variance (ANOVA) followed by a Bonferroni post-test to compare replicate means in each time point | Time 0: *p=*0.0492  Time 30’: *p=*0.0477 Time 120’: *p=*0.0464 |
| Figure 3D | Adipo-rtTA: 10  Adipo-ANG2: 10 | Two-way analysis of variance (ANOVA) followed by a Bonferroni post-test to compare replicate means in each time point | Time 30’: *p=*0.0427 Time 120’: *p=*0.0427 |
| Figure 3E | Adipo-rtTA: 10  Adipo-ANG2: 10 | Unpaired *Student’s t* test | VO_2_/Light Cycle: *p=*0.0060  VO_2_/Dark Cycle:  *p=*0.0004  VCO_2_/Light Cycle: *p=*0.0061  VCO_2_/Dark Cycle:  *p=*0.0008 |
| Figure 3F | Adipo-rtTA: 10  Adipo-ANG2: 10 | Unpaired *Student’s t* test | NEFA: *p=*0.0171  Cholesterol: *p=*0.0491 |
| Figure 3G | Adipo-rtTA: 10  Adipo-ANG2: 10 | Two-way analysis of variance (ANOVA) followed by a Bonferroni post-test to compare replicate means in each time point | 1h: *p*=0.0306  2h: *p*=0.0298 |
| Figure 3H | Adipo-rtTA: 10  Adipo-ANG2: 10 | Unpaired *Student’s t* test | Plasma: *p=*0.0046  sWAT: *p=*0.0286 |
| Figure 3I | Adipo-rtTA: 10  Adipo-ANG2: 10 | Unpaired *Student’s t* test | *Angptl3*: *p=*0.0303  *Angptl8*: *p=*0.0383 |
| Figure 3J | Adipo-rtTA: 3  Adipo-ANG2: 3 | Unpaired *Student’s t* test | *p=*0.0443 |
| Figure 4D | Adipo-rtTA: 10  Adipo-ANG2: 10 | Unpaired *Student’s t* test | *Itgam: p=*0.0071  *Ly6c1: p=*0.0276 |
| Figure 4E | Adipo-rtTA: 10  Adipo-ANG2: 10 | Unpaired *Student’s t* test | *Clec10a: p=*0.0051  *Tnfa: p=*0.0495  *Nos2: p=*0.0471 |
| Figure 4F | Adipo-rtTA: 3  Adipo-ANG2: 3 | Unpaired *Student’s t* test | sWAT: *p=*0.0495  eWAT: *p=*0.1189  *Nos2: p=*0.0471 |
| Figure 4H | Adipo-rtTA: 10  Adipo-ANG2: 10 | Unpaired *Student’s t* test | sWAT*: p=*0.9726 eWAT*: p=*0.8977  (n.s.) |
| Figure 5A | Control IgG: 6  Anti-ANG2: 5 | Unpaired *Student’s t* test | *Pecam1: p=*0.0485  *Tek: p=*0.0277 |
| Figure 5C | Control IgG: 5  Anti-ANG2: 5 | Unpaired *Student’s t* test | *p<*0.0001 |
| Figure 6D | Control IgG: 5  Anti-ANG2: 5 | Unpaired *Student’s t* test | *Il6: p=*0.0019  *Adgre1: p=*0.0445  *Tgfb1: p=*0.0436 *Col1a1: p=*0.0324  *Col6a1: p=*0.0485 |
| Figure 6E | Control IgG: 5  Anti-ANG2: 5 | Unpaired *Student’s t* test | *Ifng: p=*0.0319  *Tnfa: p=*0.0021  *Nos2: p=*0.0018 |
| Figure 6F | Control IgG: 3  Anti-ANG2: 3 | Unpaired *Student’s t* test | sWAT: *p=*0.1005  eWAT: *p=*0.0296 |
| Figure 7B | Control IgG: 5  Anti-ANG2: 5 | OGTT: Two-way analysis of variance (ANOVA) followed by a Bonferroni post-test to compare replicate means in each time point  Area Under Curve: Unpaired *Student’s t* test | Time 30’: *p=*0.0378 Area Under Curve: *p=*0.0393 |
| Figure 7C | Control IgG: 5  Anti-ANG2: 5 | ITT: Two-way analysis of variance (ANOVA) followed by a Bonferroni post-test to compare replicate means in each time point  Area Under Curve: Unpaired *Student’s t* test | Time 60’: *p=*0.0473  Time 90’: *p=*0.0369 Area Under Curve: *p=*0.0498 |
| Figure 7D | Control IgG: 5  Anti-ANG2: 5 | Two-way analysis of variance (ANOVA) followed by a Bonferroni post-test to compare replicate means in each time point | *p=*0.0036 |
| Figure 7E | Control IgG: 5  Anti-ANG2: 5 | Unpaired *Student’s t* test | Liver Triglyceride:  *p=*0.0365 |
| Figure 7F | Control IgG: 5  Anti-ANG2: 5 | Unpaired *Student’s t* test | *Angptl3*: *p<*0.0001  *Angptl4*: *p<*0.0001  *Angptl8*: *p<*0.0001 |
| Figure 7G | Control IgG: 5  Anti-ANG2: 5 | Unpaired *Student’s t* test | *p=*0.0036 |
| Figure 1-S1A | Static: 6  Run: 6 | Unpaired *Student’s t* test | *p=*0.0325 |
| Figure 1- S1B | RT: 6  Cold: 6 | Unpaired *Student’s t* test | BAT: *p=*0.0227 |
| Figure 1- S1C | Chow diet: 6  HFD: 6 | Unpaired *Student’s t* test | sWAT: *p=*0.0470 |
| Figure 1-S1D | Fed: 6  Fasting 24h: 6 | Unpaired *Student’s t* test | *Angpt2*: *p=*0.0307 |
| Figure 1-S1E | Chow diet: 6  HFD: 6 | Unpaired *Student’s t* test | sWAT Adipocytes: *p=*0.1019  sWAT SVF: *p=*0.0184 |
| Figure 2-S1A | Adipo-rtTA: 3  Adipo-ANG2: 3 | Unpaired *Student’s t* test | *p=*0.0011 |
| Figure 2-S1B | Adipo-rtTA: 10  Adipo-ANG2: 10 | Unpaired *Student’s t* test | *p=*0.0003 |
| Figure 2-S1C | Adipo-rtTA: 10  Adipo-ANG2: 10 | Unpaired *Student’s t* test | *p=*0.0053 |
| Figure 3-S2A | Adipo-rtTA: 6  Adipo-ANG2: 6 | Unpaired *Student’s t* test | Day 3:*p=*0.0057 Day 10: *p=*0.0086 |
| Figure 3-S2C | Adipo-rtTA: 6  Adipo-ANG2: 6 | Two-way analysis of variance (ANOVA) followed by a Bonferroni post-test to compare replicate means in each time point | 15’ :*p=*0.0228  30’: *p=*0.0010  60’: *p<*0.0001  120’: *p=*0.0199 |
| Figure 4-S1B | Adipo-rtTA: 10  Adipo-ANG2: 10 | Unpaired *Student’s t* test | *Itgam: p=*0.0017  *Ly6c1: p=*0.0615 |
| Figure 4-S1C | Adipo-rtTA: 10  Adipo-ANG2: 10 | Unpaired *Student’s t* test | *Mrc1: p=*0.1019  *Clec10a: p=*0.0048 |
| Figure 4-S3B | Adipo-rtTA: 10  Adipo-ANG2: 10 | Unpaired *Student’s t* test | *Col1a1: p=*0.0141  *Col3a1: p=*0.0004  *Col6a1: p=*0.0114  *Lox: p=*0.0041 |
| Figure 4-S4A | Adipo-rtTA: 10  Adipo-ANG2: 10 | Unpaired *Student’s t* test | *Vegfa: p=*0.0071  *Ucp1: p=*0.0034 |
| Figure 6-S1C | Control IgG: 5  Anti-ANG2: 5 | Unpaired *Student’s t* test | *Il10: p=*0.0247  *Tnfa: p=*0.0405  *Nos2: p=*0.0346 |
